# Hemoadhican-based self-leveling Janus patch for comprehensive prevention of postoperative adhesions

**DOI:** 10.1016/j.mtbio.2025.102021

**Published:** 2025-06-24

**Authors:** Rui Fang, Ning Yu, Simeng Chen, Xi Xu, Jianfa Zhang

**Affiliations:** a Center for Molecular Metabolism, Nanjing University of Science & Technology, Nanjing, 210094, China; b Key Laboratory of Metabolic Engineering and Biosynthesis Technology, Ministry of Industry and Information Technology, Nanjing, 210094, China

**Keywords:** Postoperative abdominal adhesions, Janus patch, Hemoadhican, Anti-adhesion

## Abstract

Postoperative anti-adhesion materials face significant challenges, including dislocation, the induction of nonspecific tissue adhesions, and secondary fibrinolytic disorders. In this study, we developed a self-leveling, transient, unilaterally bonded Janus patch (J-HD) based on hemoadhican (HD). This patch consists of three distinct functional layers: an anti-adhesion layer, a reinforcement layer, and a wet tissue adhesion layer. Each layer serves a specific purpose: the bottom anti-adhesion layer, composed of HD and Pluronic F127 (PF127), effectively prevents cells, proteins, and tissues from adhering; the middle polyvinyl alcohol (PVA) reinforcement layer enhances the mechanical properties of the film, exhibiting a tensile strength of approximately 2.02 MPa; and the top HD adhesion layer provides strong adhesion to wet tissue with an interfacial toughness of approximately 223.85 J/m^2^. The J-HD patches demonstrate excellent self-leveling ability, unilateral adhesion properties, and effective resistance to fibrin and cell adhesion *in vitro*. In a rat cecum-abdominal wall adhesion model, the J-HD patch exhibited a significant reduction in abdominal adhesions compared to commercially available Interceed® films. Mechanistically, the J-HD patch effectively regulates the fibrinolytic balance by modulating the levels of tissue plasminogen activator (t-PA) and plasminogen activator inhibitor-1 (PAI-1), promoting a nonadherent state, reducing excessive inflammatory responses, and facilitating the repair of intestinal wall integrity. *In vivo* implantation of the J-HD patch does not exhibit significant acute or chronic toxicity, elicits a favorable host response, and demonstrates biocompatibility. This study presents a potential strategy for the clinical design of Janus patches with anti-migratory and anti-adhesive properties.

## Highlights

• A Janus patch based on hemoadhican reduces postoperative abdominal adhesions.
• The patch exhibits self-leveling and excellent mechanical properties.
• J-HD patch repairs intestinal walls and balances the fibrinolytic system.
• The excellent anti-adhesion efficiency is proved in a rat abdominal adhesion model.
• The patch demonstrates good *in vitro* and *in vivo* biosafety and biodegradability.

## Graphical abstract

**Figure:**
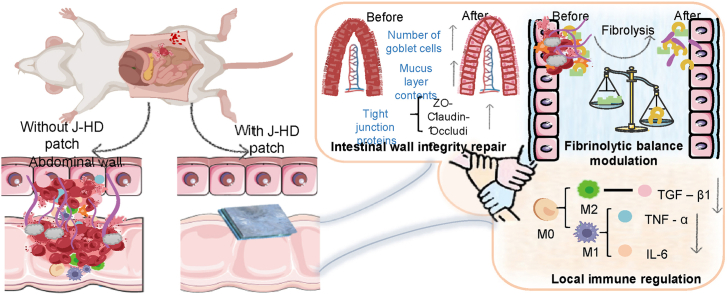

## 1 Introduction

Postoperative abdominal adhesions (PPA) are a prevalent and significant clinical issue primarily resulting from trauma and abdominal or pelvic surgeries. They can develop in over 90 % of patients following a cesarean section, leading to various complications, including intestinal obstruction, abdominal pain, and female infertility [[1], [2], [3]]. Abdominal adhesions typically arise from peritoneal injury and are exacerbated by local repair processes that involve multiple complex cascades of inflammatory responses and fibrin deposition [4,5], as well as further disruption of the balance between fibrin production and degradation [6]. These factors contribute to impaired intestinal wall integrity [7], infiltration of inflammatory cells, and excessive release of inflammatory mediators [8], ultimately resulting in the formation of adhesions. A second operation, known as adhesion release, is currently the only effective treatment for eliminating adhesions. However, approximately 80 % of patients face a significant risk of developing more severe recurrent adhesions and other medical complications postoperatively [9,10]. This underscores the urgent need for effective strategies to prevent adhesion formation.

Currently, pharmacological treatments and biomaterial barriers, along with advancements in surgical techniques, are the most common proactive measures for preventing PPA [11]. However, local or systemic pharmacological treatments, including anti-inflammatory drugs and anticoagulants, can have a failure rate of up to 85 % due to limitations such as inadequate spatial targeting and rapid clearance by the reticuloendothelial system [12]. Consequently, physical barrier materials (e.g., polymer solutions, hydrogels, and solid films) that create a physical separation between damaged tissue surfaces and surrounding tissues or organs are considered more effective adjuncts for preventing adhesions [[13], [14], [15]]. Liquid barriers formed by Icodextrin solution (Adept®) provide several advantages, including high fluidity and extensive coverage. However, they are easily diluted by intraperitoneal fluid, rapidly absorbed postoperatively, and do not remain on the wound for a sufficient duration [10,16]. Commercially available solid membrane barriers, such as Interceed® and Seprafilm®, are anti-adhesion materials composed of modified natural polymers (e.g., carboxymethylcellulose) and are widely recognized as the clinical gold standard for preventing postoperative adhesions [17,18]. While these films have a significantly longer degradation time and have demonstrated approximately 32–55 % effectiveness in clinical trials [19,20], their lack of self-adhesive properties necessitates the use of surgical sutures [21]. This requirement can lead to secondary injuries and limits complete coverage of irregular wounds, thereby increasing the risk of barrier migration. Sodium hyaluronate gel can effectively cover irregular wounds; however, its rapid degradation (with a half-life of approximately 24 h), low mechanical strength, and poor adhesion to the target tissue hinder its effectiveness during the critical phase of adhesion formation [22,23]. As a result, the clinical efficacy of currently available commercial products in preventing adhesions is significantly lower than anticipated.

Achieving strong adhesion to wet tissues is often challenging due to the constantly dynamic *in vivo* environment, where wet tissue surfaces and various organs frequently interact. The hydration layer acts as a non-selective interfacial barrier that impedes adhesion between the adhesive and the target tissue [24,25]. Consequently, the material must demonstrate not only robust adhesion to wet tissue defects but also resistance to adhesion with surrounding healthy tissues [26]. Many researchers have investigated adhesive hydrogel films with Janus properties for the prevention of PPA, as these materials can mitigate secondary injuries and tissue adhesions caused by suturing. Some strategies focus on creating a hydrogel barrier through *in situ* covalent cross-linking of precursor solutions; however, these adhesive films often lack the mechanical strength and anti-inflammatory properties necessary for effective PPA repair [[27], [28], [29]]. Furthermore, these hydrogel precursors can be easily diluted by blood or bodily fluids and typically require auxiliary ultraviolet light for gelation, which complicates the procedure [30]. Additionally, most adhesive hydrogels are irreversible and challenging to remove once adhered to tissues, and their prolonged *in vivo* persistence without degradation may increase associated risks [1,31]. Therefore, the development of degradable, multifunctional anti-adhesion films with Janus adhesion properties and asymmetric side-specific functionality is essential. These films must effectively prevent postoperative tissue adhesion and secondary injury by modulating inflammatory responses and cellular behaviors. Additionally, they should possess excellent mechanical and self-leveling properties while providing controllable unilateral adhesion, in contrast to conventional Janus patches [32].

**Fig. 1:**
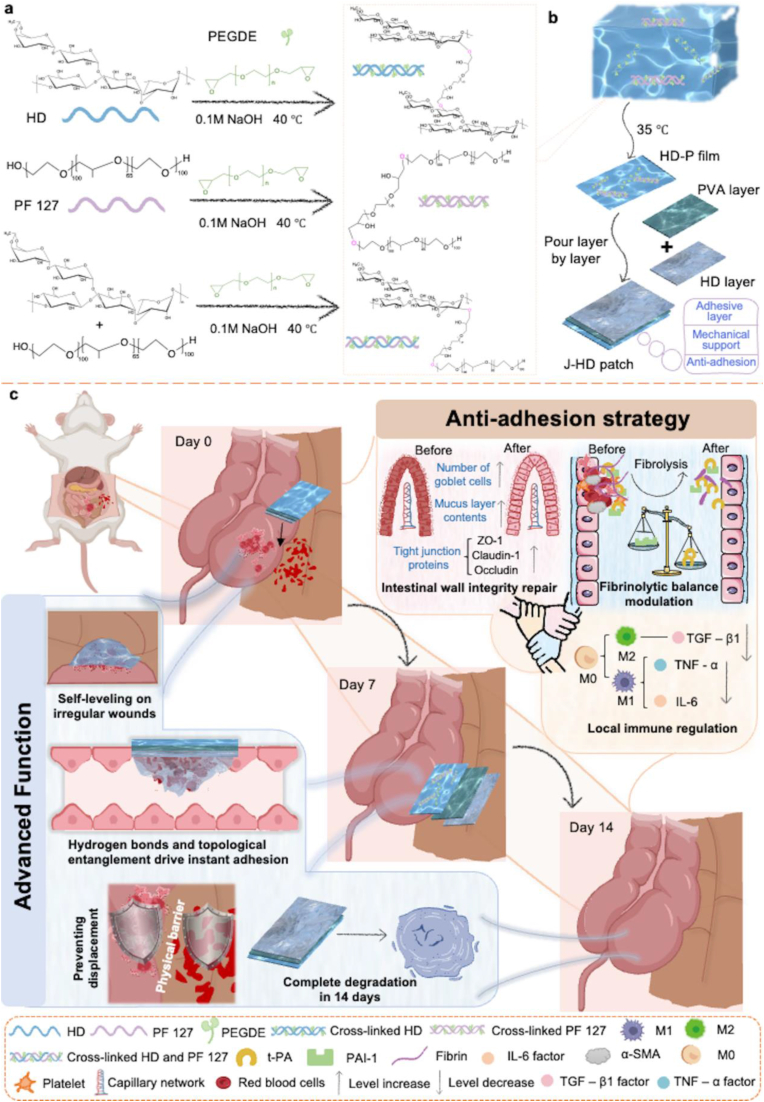
Synthesis and anti-adhesion therapeutic evaluation of J-HD patches. (a) The reaction formula for cross-linking HD polysaccharides and PF127 with PEGDGE at 40 °C in an alkaline environment. (b) Synthesis of the J-HD patch. (c) The anti-adhesion therapeutic effects of J-HD patches in a rat cecum-peritoneal cavity model following surgery.

Previous studies have demonstrated that *Paenibacillus* sp. 1229 produces an extracellular polysaccharide known as hemoadhican (HD), which features a hexasaccharide repeating unit with the following structure: →)-α-L-Rhap-(1 → 3)-β-D-Glcp-(1 → 4)[4,6-ethylidene-α-D-Galp-(1 → 4)-α-D-Glcp-(1 → 3)]-α-D-Manp-(1 [33]. This polysaccharide exhibits immediate and exceptionally strong adhesion to wet tissues through extensive hydrogen bonding and topological entanglement, surpassing most reported polysaccharide-based adhesives, such as photocrosslinked hyaluronic acid (HA) hydrogels (approximately 13 kPa) and carboxymethyl chitosan/dextran/poly (ionic liquid)-based hydrogel (approximately 12 kPa) [34,35]. In comparison to these studies, the adhesion performance of HD polysaccharide sponges achieved an impressive adhesion strength of approximately 24.9 kPa, indicating a significant tissue adhesion potential [36,37]. Remarkably, upon cross-linking with polyethylene glycol diglycidyl ether (PEGDE), HD retains its bioactivity and demonstrates excellent efficacy in preventing postoperative abdominal adhesions [38]. Pluronic F127 (PF127) is an FDA-approved, biocompatible amphiphilic polymer that is widely utilized in biomedical applications. It consists of two hydrophilic poly (ethylene oxide) (PEO) blocks flanking a central hydrophobic polypropylene oxide (PPO) block [39], making it an excellent hydrogel matrix. In this study, we designed a self-leveling, unilaterally adhesive three-layer Janus patch (J-HD). This patch consists of a top HD adhesion layer, an intermediate polyvinyl alcohol (PVA) reinforcement layer, and a bottom HD-PF127 anti-adhesion layer. It achieves multiple synergistic effects, including anti-adhesion, mechanical reinforcement, and strong adhesion to wet tissue. Specifically, PF127 and HD were combined to create a base layer (HD-P) with superior antifouling properties (Fig. 1a). The intermediate layer consisted of a PVA film that exhibited high toughness and elasticity, while the top adhesive layer was composed of a Janus patch (J-HD) derived from HD (Fig. 1b). In this study, the *in vitro* properties of J-HD patches were validated, including their anti-fibrin adhesion, anti-cellular adhesion, and transient unilateral adhesion capabilities. Furthermore, their *in vivo* efficacy in modulating non-adherent fibrinolytic homeostasis following surgical procedures was confirmed using an intraperitoneal rat model, along with an assessment of their biocompatibility (Fig. 1c). The combination of strong wet-tissue adhesion, anti-post-operative adhesion capabilities, and excellent biocompatibility in J-HD patches provides valuable insights for the development of novel anti-adhesion materials.

## 2 Materials and methods

### 2.1 Materials and reagents

The HD polysaccharide (molecular weight = 23700 kDa) was extracted and purified from the fermentation broth following the procedures outlined in our previous publication [30]. Polyvinyl alcohol (PVA-1799, Mw: 74800, 98–99 % hydrolyzed) and PEGDE (epoxy value: 0.70–0.80 mol/100 g) were purchased from Aladdin, China. Glycerol and fibrinogen were obtained from Sigma-Aldrich (Shanghai, China). Pluronic F127, the BCA Protein Concentration Determination Kit, the CCK-8 Assay Kit, and the Calcein/PI Cell Viability/Cytotoxicity Assay Kit were supplied by Beyotime Biological Reagent Co., Ltd. (Shanghai, China). ELISA kits were acquired from Shanghai Enzyme-linked Biotechnology Co., Ltd. (Shanghai, China). Fresh pork skin was sourced from the local market. L929 mouse fibroblasts and RAW264.7 mouse mononuclear macrophage cells were obtained from the China Center for Type Culture Collection, while blood cells were collected from normal Sprague-Dawley (SD) rats. All reagents were of analytical grade and used as received, and all water used in the experiments was deionized. Male SD rats aged between 8 and 10 weeks, weighing 220 ± 20 g, were utilized. All animal experiments complied with the ARRIVE guidelines and were approved by the Institutional Animal Care and Use Committee at Nanjing University of Science and Technology (IACUC-NJUST-2023-0610).

### 2.2 Preparation of HD-P films

According to the published protocol with minor modifications [38], 0.16 g of HD powder and varying amounts of PF127 (0, 2, 4, and 6 mg) were completely dissolved in an aqueous NaOH solution (0.1 M). Subsequently, PEGDE was added to the HD solution while stirring at a ratio of 1:0.072 (v/v), and the reaction mixture was incubated at 40 °C for 12 h. The mixture was then neutralized with 0.1 M HCl. The resulting hydrogels were thoroughly washed 12 to 15 times with distilled water to remove any residual unreacted chemicals. Finally, the hydrogels were cast into molds and dried in an oven at 35 °C to produce the anti-adhesion low-layer films designated as HD-P_0_, HD-P_L_, HD-P_M_, and HD-P_H_.

### 2.3 Preparation of HD films

HD films were prepared using varying concentrations of polysaccharide HD (2 %, 4 %, and 6 %). First, HD was dissolved in double-distilled water at 95 °C while being continuously stirred and subjected to ultrasonication for 2 h. The HD solutions were then centrifuged at 9000 rpm for 3 min to eliminate any undissolved particles. After centrifugation, the supernatants were transferred to molds and dried at 60 °C to produce the desired HD film products.

### 2.4 Preparation of Janus patch

The J-HD patch consists of three layers: a dry hydrogel HD-P base layer, an intermediate layer of PVA hydrogel, and a top layer of HD film, which is applied to one side of the PVA plate. The microcrystalline cross-linked PVA hydrogel was prepared using three freeze-thaw cycles, as described by Lin et al. [40]. This PVA hydrogel was then deposited onto the HD film, which was prepared according to Section 2.3, to form the intermediate layer. After drying at 60 °C, the HD-P hydrogel, prepared as described in Section 2.2, was spread onto the opposite side of the PVA sheet and dried at 35 °C to yield the complete J-HD patch. For comparison, the J-HD patch without the PVA intermediate layer was prepared by directly spreading the HD-P hydrogel onto the HD film and drying it at 35 °C.

### 2.5 Physicochemical characterization of HD-P films

Fourier transform infrared spectroscopy (FTIR; Nicolet iS20, Thermo Fisher Scientific) was conducted in the range of 525–4000 cm^−1^ to analyze the chemical bonds present in the HD-P films. X-ray diffraction (XRD) analysis was performed using a D8-ADVANCE diffractometer (Bruker, Germany) with a 2θ scanning range of 5–90° to investigate the crystalline structure. Prior to surface morphology analysis, the HD-P samples were sputter-coated with gold. The surface morphology was then examined using a field-emission scanning electron microscope (SEM; Sigma 300, ZEISS, Germany). Following the method described by Wan et al., [30] the mechanical properties of the HD-P films were evaluated using an electronic universal testing machine (ETN503A, WANCE, China). HD-P films (2 × 2 cm) were stretched at a constant rate of 10 mm/min until fracture, and the corresponding stress–strain curves were recorded for each sample.

### 2.6 *In vitro* degradation experiments

The *in vitro* degradation behavior of the HD-P films was assessed by monitoring changes in sample weight over time during immersion in phosphate-buffered saline (PBS, pH 7.4). A known initial weight of HD-P was immersed in 10 mL of PBS at 37 °C. At predetermined time points, samples were removed, dried, and weighed, while the PBS solution was refreshed daily. The degradation kinetics of the HD-P films were calculated by Eq. (1).

(1) $$Weight\;loss=\frac{m_{0}-m_{t}}{m_{0}}\times 100\;\%$$

where m_0_ is the initial dry weight of the HD-P sample, and m_t_ is the remaining dry weight at a specified time point.

### 2.7 X-ray Photoelectron Spectroscopy

HD-P films were attached to the sample stage using conductive adhesive, and their surface chemical composition was analyzed with an ESCALAB Xi^+^ electron spectrometer (Thermo Fisher Scientific, USA).

### 2.8 Interfacial toughness of HD on wet tissue

The interfacial toughness of HD on wet tissue was evaluated using a 180° peel test conducted at a speed of 50 mm/min, in accordance with ASTM standard F2256. The interfacial toughness was calculated by Eq. (2).

(2) $$Interfacial\;toughness=\frac{2F}{W}$$

Where F represents the steady-state plateau force, W denotes the overlap width of the HD sample (n = 3).

### 2.9 Overlap shear test

Wet pork skin samples were preheated to 37 °C for 30 min prior to testing. The HD samples were applied to the tissue surfaces following in accordance with standard F2255, and the overlap shear test was performed conducted a rate of 50 mm/min. Shear strength was calculated by dividing the maximum force by the contact area (30 mm in length and 10 mm in width). Lap shear strength was determined using Eq. (3).

(3) $$Shear\;strength=\frac{F}{A}$$

Where F represents the maximum load recorded, and A denotes the adhesion area between the HD and the tissue (n = 3).

### 2.10 Characterization of the basic physicochemical properties of J-HD patches

The thermal decomposition behavior of the J-HD patches was analyzed using a thermogravimetric analyzer/differential scanning calorimeter (TGA/DSC 1/1100LF, METTLER TOLEDO, Switzerland) over a temperature range of 25 °C–800 °C. Mechanical properties were evaluated using an electronic universal testing machine. The surface and cross-sectional morphology of the J-HD patches were examined by scanning electron microscopy (SEM).

### 2.11 *In vitro* anti-adhesion

The *in vitro* anti-adhesion performance of J-HD patches was evaluated using previously established methods [38,41]. In a 24-well cell culture plate, each well was individually coated with either the top or bottom surface of a sterilized J-HD patch. Subsequently, 2 mL of fresh medium containing L929 fibroblasts (3 × 10^4^ cells/well) was added to each well. Cells cultured directly on tissue culture polystyrene (TCP) served as the control group. After 24 h of incubation, the cells were gently washed three times with PBS and stained using Calcein-AM/PI dye. Cell attachment on the surface of the J-HD patches was visualized using inverted fluorescence microscopy (Ti2, Nikon, Japan).

To evaluate protein and cell adhesion, the top and bottom surfaces of J-HD patches were incubated with fluorescein isothiocyanate (FITC)-labeled fibrinogen (2 mg/mL) or with blood cells at 37 °C for 2 h. After incubation, the surfaces were gently rinsed three times with PBS to remove loosely adsorbed proteins or cells. The remaining adsorbed proteins or blood cells were observed using inverted fluorescence microscopy. The same procedure was employed to assess blood cell adhesion on the J-HD surfaces.

### 2.12 Self-leveling behavior

To evaluate the self-leveling properties of the J-HD patch, a J-HD patch was placed flat on the surface of a steel bead, and 200 μL of PBS was added dropwise. The film's response and behavior upon contact with PBS were observed visually.

### 2.13 *In vitro* anti-inflammatory activity

The anti-inflammatory activity of the J-HD patches was evaluated using the method described by Zhang et al. [42]. RAW 264.7 macrophages were seeded in 6-well plates at a density of 2 × 10^5^ cells per well and incubated at 37 °C in a humidified atmosphere containing 5 % CO_2_ to allow facilitate attachment. Sterilized J-HD patches (6 mg) were immersed in 6 mL of DMEM for 48 h to prepare the extract. After incubation, the films were removed, and the extract was collected for subsequent experiments.

To induce M1 polarization, RAW 264.7 cells were stimulated with lipopolysaccharide (LPS, 1 μg/mL) and interferon-γ (IFN-γ, 40 ng/mL) in the presence of the J-HD patch extract (final concentration: 1 mg/mL) for 24 h. The anti-inflammatory effect of the J-HD patch extract was assessed by measuring evaluated concentrations of tumor necrosis factor-α (TNF-α), interleukin-6 (IL-6), and transforming growth factor-β1 (TGF-β1) in the culture supernatant using enzyme-linked immunosorbent assay (ELISA) kits, following the manufacturers’ protocols. **Untreated RAW 264.7** cells served as the control group.

### 2.14 *In vivo* anti-adhesion evaluation

The *in vivo* anti-adhesion effect of J-HD patches was evaluated using a rat cecum–abdominal wall adhesion model, as previously reported [43]. A total of 48 male SD rats were randomly divided into four groups (n = 12 per group): sham-operated, model, Interceed®, and J-HD groups. After fasting for 8 h and withholding water for 2 h prior to surgery, the rats were anesthetized with an intraperitoneal injection of 1 % sodium pentobarbital (40 mg/kg). The abdominal hair was shaved and disinfected. A 2.5 cm midline incision was made to expose the cecum, and the cecal surface was gently rubbed with sterile dry gauze until pinpoint bleeding occurred. A 1 cm × 2 cm defect was then created in the corresponding area of the abdominal wall, and the wounds on both sides were partially sutured using 3-0 silk sutures to induce adhesions. In the sham-operated group, only cecal exposure to air for 5 min was performed, followed by irrigation with 200 μL of saline. In the model group, 200 μL of physiological saline was applied to the injured cecum before closure. In the Interceed® and J-HD groups, an Interceed® film or J-HD patch was applied, respectively, before closing the incision with 4-0 silk sutures in both the muscle and skin layers. All surgical procedures were performed under aseptic conditions and supervised by an experienced surgeon. Postoperative recovery was monitored by measuring body weight to assess gastrointestinal motility. On postoperative days 7 and 14, the rats were euthanized via intraperitoneal injection of 0.2 % sodium pentobarbital, and the severity of intra-abdominal adhesions was evaluated and photographed. Adhesion severity was assessed in a double-blind manner according to a standardized adhesion scoring system (Table S1) [44]. Blood was collected from the tail vein for routine hematological analysis, and relevant tissues were harvested for histological examination using hematoxylin and eosin (H&E) and Masson's trichrome staining. Furthermore, reverse transcription polymerase chain reaction (RT-PCR) was employed to quantify the mRNA expression levels of tissue plasminogen activator (t-PA), plasminogen activator inhibitor-1 (PAI-1), collagen type I (Col-1) and α-smooth muscle actin (α-SMA) in the collected tissues.

### 2.15 Observation and analysis of cecum wall integrity

Structural changes at the injured sites in each group were assessed using H&E staining, Masson's trichrome staining, periodic acid-Schiff (PAS) staining, and Alcian blue (AB) staining. Additionally, the expression levels of tight junction proteins, including ZO-1, claudin-1, and occludin, were quantified at the gene level using RT-qPCR. GAPDH was used as the internal reference gene, and all RT-qPCR primer sequences are listed in Table S2.

### 2.16 Immunofluorescence analysis

Immunofluorescence staining was conducted to evaluate the phenotypic shift of macrophages from M1 to M2 on postoperative day 7. The stained tissue sections were observed under a fluorescence microscope, and digital images were captured with a PANNORAMIC MIDI II scanner. Fluorescence intensity was analyzed using ImageJ software to assess the extent of macrophage polarization.

### 2.17 *In vitro* cytotoxicity evaluation

Hematocompatibility was evaluated by measuring the release of hemoglobin from erythrocytes to determine the hemolysis rate of the material, following the method described by Liu et al. [45]. The cytotoxicity of the J-HD patch was assessed by measuring the growth and viability of L929 cells using the CCK-8 assay kit and the calreticulin/PI cell viability/cytotoxicity assay kit, in accordance with the manufacturer's instructions.

### 2.18 *In vivo* degradation

The biocompatibility of the J-HD patch was further assessed through *in vivo* degradation experiments [46,47]. Six SD rats were randomly divided into two groups: the trauma group and the J-HD patch group, with three rats in each group. The rats were anesthetized and positioned on a flat surface. Their backs were shaved and sterilized with iodophor. A sterile scalpel was used to create a longitudinal incision, and the J-HD patch was inserted subcutaneously in the J-HD group, while the trauma group received only the incision as a control. After two weeks, the rats were euthanized and tissue samples around the encapsulated material and major organs (heart, liver, spleen, lungs, kidneys and bladder) were collected for histological evaluation. A 200 μL blood sample was obtained from each rat for blood examination.

### 2.19 Statistics and analysis

All experiments were conducted in triplicate or more, and the data are presented as the mean ± standard deviation (SD). Statistical analyses were performed using GraphPad Prism 9 and ImageJ software. Statistical significance was assessed using Student's *t*-test. For comparisons involving more than two groups, one-way ANOVA was employed, followed by Tukey's *post hoc* test. Differences were considered statistically significant at *p* < 0.05 (∗), *p* < 0.01 (∗∗), and *p* < 0.001 (∗∗∗), while ns indicates no significant difference.

## 3 Results and discussion

### 3.1 Synthesis and physicochemical properties of HD-P films

**Fig. 2:**
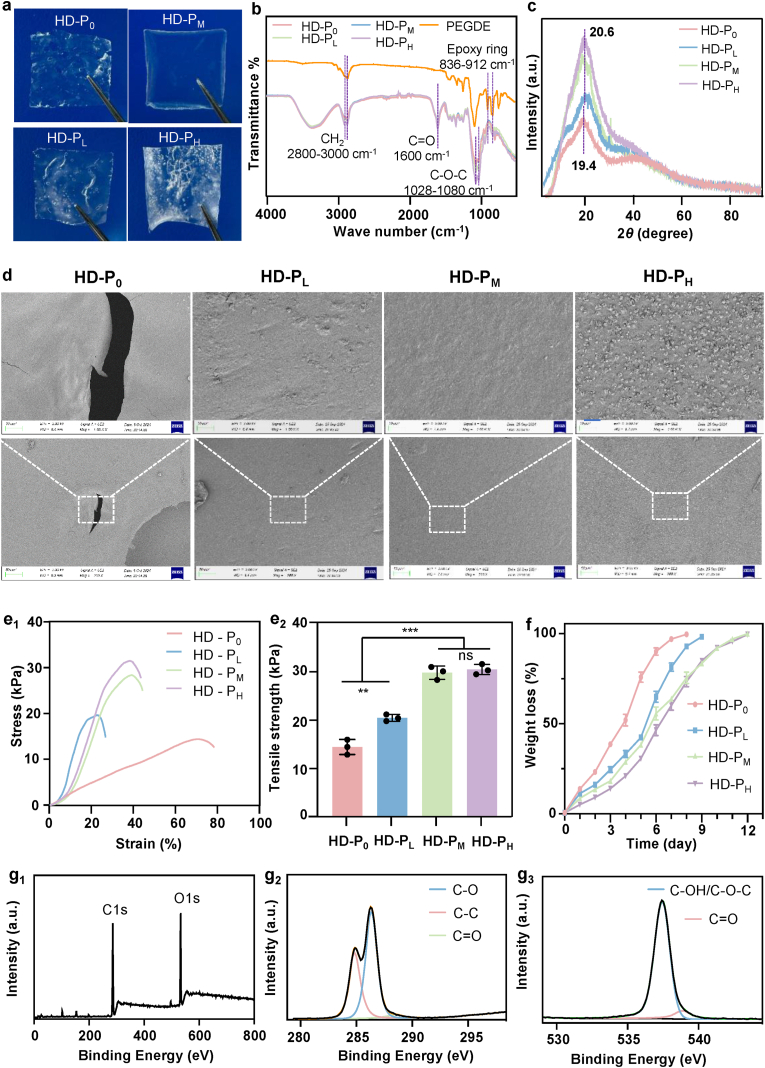
Characterization of HD-P films. HD-P_0_, HD-P_L_, HD-P_M_, and HD-P_H_ are anti-adhesion base films prepared with 0, 2, 4, and 6 mg of PF127, along with 0.16 g of HD, respectively. (a) Macroscopic image, (b) fourier transform infrared spectroscopy (FTIR) spectra, (c) X-ray diffraction (XRD) spectra, and (d) scanning electron microscope (SEM) images of HD-P films. (e_1_) Strain–stress curves and (e_2_) tensile strength of HD-P films from tensile tests (n = 3). (f) Spreadability measurements of HD-P films (n = 3). (g_1_) X-ray Photoelectron Spectroscopy (XPS) full survey of the HD-P_M_ film, along with high-resolution XPS data for (g_2_) C1s and (g_3_) O1s of the HD-P_M_ film. Statistical significance analysis was evaluated using one-way ANOVA with ∗∗*p* < 0.01 and ∗∗∗*p* < 0.001. Error bars show standard deviation, ns indicates no significant difference.

To fabricate Janus patches, a layer-by-layer assembly strategy was employed. The antifouling layer was prepared by crosslinking HD with PF127 using PEGDE as a crosslinking agent. As shown in Fig. 2a, the HD-P_0_ film exhibited numerous irregularly distributed pores. With increasing PF127 content, the film surface became denser. Although bubbles and pores remained visible on the surface of the HD-P_L_ film, the HD-P_M_ film displayed a smoother and more compact morphology. In contrast, the surface of the HD-PH film appeared relatively rough, with the presence of white spots. Furthermore, FTIR was utilized to analyze the chemical structures of PEGDE, HD-P_0_, HD-P_L_, HD-P_M_, and HD-P_H_ films in order to assess the degree of crosslinking (Fig. 2b). The FTIR spectra were normalized based on the intensity of the carbonyl peak at 1600 cm^−1^ [48,49]. Characteristic peaks were observed in the range of 2800–3000 cm^−1^, corresponding to symmetric and asymmetric CH_2_ stretching vibrations, along with a peak at 1080 cm^−1^ attributed to the C–O–C stretching vibration [50]. In the spectra of HD-PF_L_, HD-PF_M_, and HD-PF_H_, the intensity of these peaks increased with the incorporation of PF127 compared to the HD spectrum, indicating the successful introduction of EG repeating units through ether crosslinking between the hydroxyl and epoxy groups from PEGDE and PF127. Peaks at 912 and 836 cm^−1^, which are characteristic of the epoxy rings in PEGDE, were absent in the HD-P spectra, indicating the consumption of epoxy rings during the crosslinking reaction [51].

The broad amorphous peak observed in the XRD pattern of the HD-P film indicates its non-crystalline nature (Fig. 2c). The presence of amorphous regions enhances the water-holding capacity of the copolymer, a desirable characteristic for polymers intended for biomedical applications [52]. A noticeable increase in crystallinity was observed in certain diffraction peaks, primarily attributed to the incorporation of PF127. Upon crosslinking with PEGDE and HD, PF127 promotes a more compact molecular arrangement compared to the relatively loose structure of HD-P_0_, thereby enhancing the regularity of the polymer chains. Notably, the diffraction peaks of HD-P_0_ and HD-P_L_ appeared at 2θ = 19.4°, while those of HD-P_M_ and HD-P_H_ shifted to approximately 20.6°, indicating that structural transformations occur with the addition of PF127. Differences in the microstructure of HD-P films across groups were further revealed by SEM imaging (Fig. 2d). The HD-P_0_ film exhibited visible pores and cracks, suggesting a loose surface network formed by HD crosslinked solely with PEGDE. In contrast, the incorporation of PF127 resulted in a denser surface morphology. The surface of HD-P_L_ remained uneven, while HD-P_H_ displayed the presence of precipitated particles. Among the samples, HD-P_M_ exhibited the smoothest and most compact surface structure, which may contribute to a reduced tendency for cell adhesion.

The incorporation of PF127 significantly enhanced the tensile strength of HD-P films, with no notable difference observed between HD-P_M_ and HD-P_H_ (14.27 ± 1.55 kPa for HD-P_0_, 20.27 ± 0.70 kPa for HD-P_L_, 29.60 ± 1.37 kPa for HD-P_M_, and 30.27 ± 1.06 kPa for HD-P_H_; Fig. 2e_1_–e_2_). Weight loss analysis indicated that HD-P_0_ was nearly completely degraded by day 8, while the degradation time was extended with the addition of PF127. Both HD-P_L_ and HD-P_M_ showed prolonged degradation periods of up to 11 days (Fig. 2f). This extended degradation time is advantageous in medical applications where the removal of implanted materials through secondary surgery is undesirable, and where the presence of anti-adhesion barriers is critical during the peak period of postoperative adhesion formation. These findings suggest that HD-P_M_ films are promising candidates for effective barriers against postoperative adhesions. Dissolution studies demonstrated that PF127 effectively inhibited the rapid dissolution of HD-P films within 24 h (Fig. S1). This effect is likely attributed to the formation of collapsed hydrophobic chain segments by PF127, which counteracts the inherent solubility of hydrogels [53]. This characteristic is beneficial for ensuring that the subsequent J-HD layer retains its adhesive strength and prevents unintended compression or damage to surrounding tissues.

Fig. 2g_1_ presents the full XPS spectrum of the HD-P_M_ film, indicating the presence of carbon (C) and oxygen (O) elements. The high-resolution C1s spectrum (Fig. 2g_2_) displays three characteristic peaks corresponding to C–C (284.8 eV), C–O (286.3 eV), and C=O (288.5 eV). Similarly, the high-resolution O1s spectrum (Fig. 2g_3_) reveals two main peaks at 531.1 eV and 532.6 eV, which are attributed to C–OH/C–O–C and C=O, respectively. These findings further confirm the successful crosslinking of PF127, HD, and PEGDE through ether bond formation, resulting in a dense polymeric network. Considering their physicochemical properties, the HD-P_0_, and HD-P_L_ films appeared porous and loose, exhibited weak mechanical strength, and demonstrated a rapid *in vitro* degradation rate. In contrast, the HD-P_M_ films displayed comparable mechanical properties, an appropriate degradation time, and a slow swelling rate, along with a smoother and denser structure than the HD-P_H_ group. Therefore, the HD-P_M_ films were selected for further experimental study.

### 3.2 Wet tissue adhesion properties of HD films

**Fig. 3:**
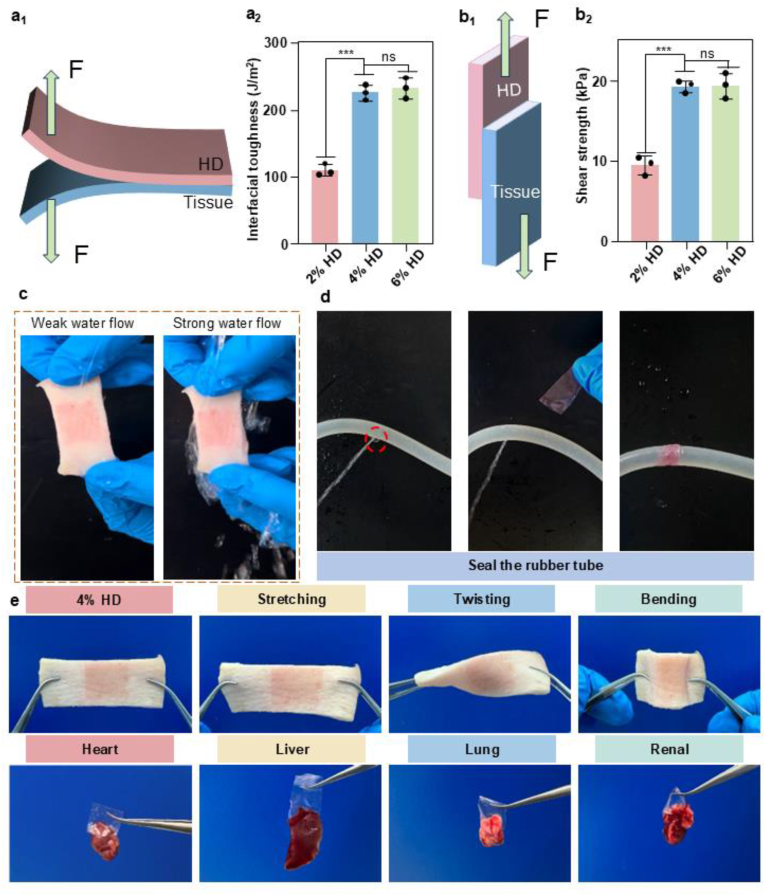
Adhesion performance of HD films. (a_1_) 180° peeling test model. (a_2_) Effect of different concentrations of HD films on interfacial toughness. (b_1_) Lap-shear model. (b_2_) Effect of different concentrations of HD films to lap-shear strength. (c) Photographs of a 4 % HD film adhering tightly to pig skin under water rinsing. (d) Efficacy of 4 % HD films in sealing leaking water mains. (e) Photograph of a 4 % HD film unilaterally adhered to porcine skin tissue and biological tissue (heart, liver, spleen, lungs and kidneys). Statistical significance analysis was evaluated using one-way ANOVA with ∗∗∗*p* < 0.001. Error bars show standard deviation, ns indicates no significant difference.

Achieving strong adhesion in wet environments remains a significant challenge [40]. The adhesion strength of HD films with varying concentrations was evaluated on wet pigskin using a 180° peel test (Fig. 3a_1_). Compared to the 2 % HD film, the 4 % HD film exhibited a significantly enhanced adhesion strength, while no statistically significant difference was observed between the 4 % and 6 % HD films. The 4 % HD film rapidly formed interfacial interactions with the moist tissue surface, achieving an interfacial toughness of approximately 223.85 ± 11.33 J/m^2^ (Fig. 3a_2_). This superior adhesion performance is attributed to abundant hydrogen bonding, topological entanglement, and chain diffusion at the interface, which synergistically reinforce interfacial adhesion [36]. In the lap-shear test, the 4 % HD film demonstrated an adhesion strength of approximately 19.16 ± 0.78 kPa on wet pigskin, significantly higher than that of the 2 % HD film (9.39 ± 1.16 kPa) and comparable to the 6 % HD film (19.27 ± 1.59 kPa) (Fig. 3b_1_–b_2_). These findings indicate that increasing the HD concentration can effectively enhance the adhesive properties of the films. Considering both performance and economic factors, the 4 % HD film was selected for subsequent experiments.

The primary cause of intraperitoneal anti-adhesion failure is the displacement of physical barriers [54]. Currently available clinical anti-adhesive materials typically lack intrinsic self-adhesive properties and are often sutured to the injured peritoneal surface. This method increases the risk of secondary trauma and re-adhesion. To address this limitation, the development of films with unilateral self-adhesive capabilities presents a promising strategy. As illustrated in Fig. 3c, the 4 % HD film remained intact and securely adhered to wet pigskin, even after exposure to water flow, demonstrating its robust wet adhesion. To further assess its sealing capability under wet conditions, a 3 mm perforation was created in the center of a rubber water hose. The 4 % HD film effectively sealed the leakage site, with no fluid leakage observed after applying brief manual pressure (Fig. 3d). Furthermore, the 4 % HD film exhibited strong adhesion to pigskin despite the application of external mechanical stresses, including stretching, twisting, and bending, and demonstrated excellent adhesive performance across various organs (Fig. 3e).

### 3.3 *In vitro* anti-adhesion efficacy of J-HD

We evaluated the anti-adhesion performance of Janus patches created by directly laminating a 4 % HD adhesive layer with an HD-P_M_ anti-adhesive film. Due to the potential for crosslinking and physical entanglement between polymer chains, precursor monomer solutions from the upper and lower layers may interdiffuse, compromising the integrity of the adhesive layer and impair the anti-adhesive properties of the opposing surface. To address this issue, PVA was introduced as a structural backbone in the film design, serving as a physical barrier that effectively prevents interlayer diffusion and preserves the cohesive integrity and adhesive strength of both layers.

**Fig. 4:**
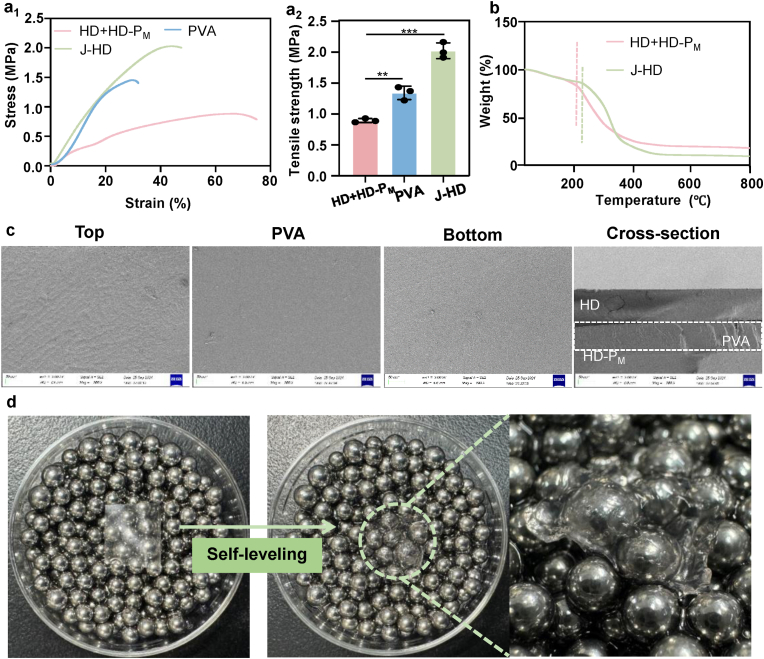
Physical and chemical properties of J-HD patches. (a_1_) Strain curve and (a_2_) tensile strength of J-HD, PVA and HD + HD-P_M_ in tensile tests (n = 3). (b) Thermogravimetric analysis (TGA) of J-HD and HD + HD-P_M_. (c) SEM image of the J-HD patch. (d) Self-leveling performance of the J-HD patch. Statistical significance was determined using one-way ANOVA with ∗∗*p* < 0.01 and ∗∗∗*p* < 0.001. Error bars show standard deviation.

Tensile testing was conducted to evaluate the mechanical toughness of the J-HD patch. The tensile strengths of the direct composite film, composed of 4 % HD and HD-P_M_ (HD + HD-P_M_), and the PVA film were measured at 0.89 ± 0.03 MPa and 1.34 ± 0.11 MPa, respectively. In contrast, the tensile strength of the J-HD patch reached 2.02 ± 0.13 MPa (Fig. 4a_1_–a_2_). The use of PVA as an intermediate isolation layer significantly enhances the tensile properties of the J-HD patch, enabling it to withstand a maximum intra-abdominal pressure of 0.02 MPa. This capability meets the mechanical requirements for tension-free abdominal wall defect repair [55]. Using HD + HD-P_M_ as the control group, we evaluated the thermal stability of the J-HD patches (Fig. 4b). Both HD + HD-P_M_ films and J-HD patches exhibited a characteristic two-stage weight loss. Below 225 °C, the initial mass loss was attributed to the evaporation of bound water in the J-HD patch [56]. A sharp mass loss occurred above 225 °C, indicating that the structural decomposition of the J-HD patch primarily takes place at elevated temperatures. Although the TGA curves of the HD + HD-P_M_ films were similar to those of the J-HD patches, the former exhibited a thermal decomposition temperature of 210 °C. This suggests that the presence of PVA enhances the thermal stability of the J-HD patch under high-temperature conditions. SEM analysis revealed that the top HD layer exhibited a rough surface morphology, which is advantageous for cell adhesion and proliferation, thereby promoting wound healing [55]. The intermediate PVA layer, characterized by its smooth structure, contributed to enhanced mechanical strength and interlayer cohesion. Meanwhile, the bottom HD-P_M_ layer featured a smooth surface that effectively reduced cell and tissue adhesion. These three layers were tightly integrated into a cohesive, sandwich-like structure (Fig. 4c). Given the irregular and heterogeneous nature of abdominal wall wounds [57], it is essential for anti-adhesion films to demonstrate self-leveling properties. As illustrated in Fig. 4d, self-leveling simulation experiments indicated that the J-HD patch was capable of filling small gaps between irregular and rounded surfaces (diameter = 10 mm) within 5 min, resulting in a smooth interface. This self-leveling capability ensures complete coverage of abdominal or intestinal wounds, thereby minimizing postoperative friction and reducing the likelihood of nonspecific adhesions.

**Fig. 5:**
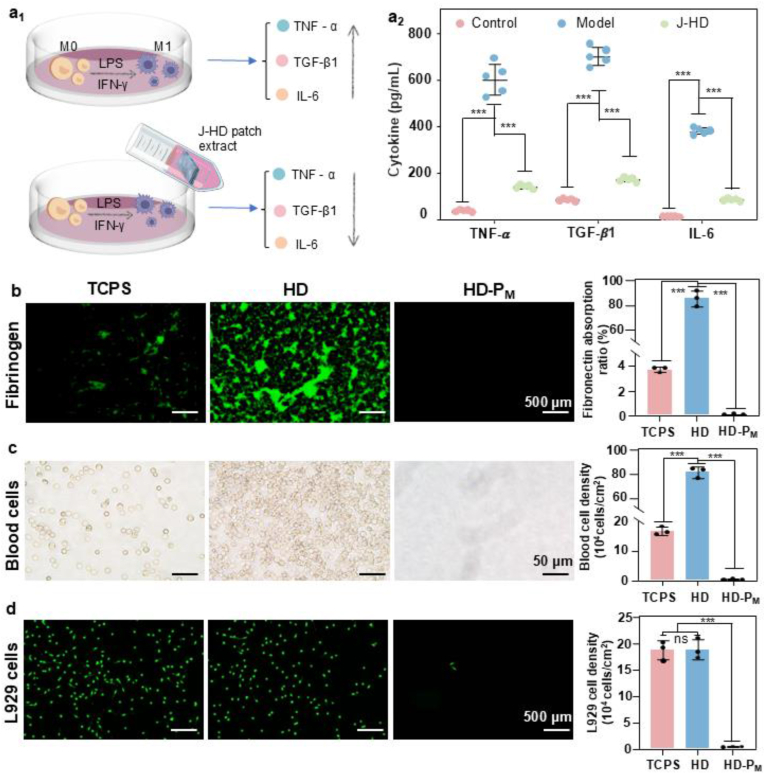
The anti-inflammatory and anti-adhesive properties of J-HD patches. (a_1_) J-HD patches effectively inhibit the secretion of pro-inflammatory cytokines by macrophages. (a_2_) The expression levels of cytokines IL-6, TNF-α, and TGF-β1 (n = 5). (b) Representative fluorescence images and quantitative analysis of fluorescently labeled fibrinogen adhesion on different films (n = 3). (c) Representative micrographs and quantitative analysis of blood cell adhesion on different films (n = 3). (d) Representative fluorescence images and quantitative analysis of L929 cell adhesion on different films (n = 3). Statistical significance was determined using one-way ANOVA with ∗∗*p* < 0.01 and ∗∗∗*p* < 0.001. Error bars show standard deviation, ns indicates no significant difference.

The critical window period for adhesion formation, driven by inflammation-induced fibrotic deposition, typically occurs within the first 7 days post-surgery [58]. Among the key immunomodulatory cytokines secreted by macrophages, TNF-α, TGF-β1, and IL-6 play pivotal roles. IL-6 contributes to adhesion formation by promoting localized inflammatory responses at wound sites [59]. TNF-α disrupts fibrinolytic homeostasis by stimulating fibroblast proliferation and enhancing the synthesis of fibrinolytic inhibitors, thereby exacerbating adhesion development [60]. TGF-β1 serves as a crucial mediator linking traumatic inflammation to fibrogenesis, promoting fibrosis through multiple signaling pathways [42]. To evaluate the anti-inflammatory properties of the J-HD patch, M0 macrophages without cytokine stimulation were utilized as the control group, while LPS and IFN-γ-treated M1 macrophages served as the inflammatory model. The concentrations of TNF-α, IL-6, and TGF-β1 in the culture supernatant were measured after exposure to J-HD patch extracts and following co-culture with M1 macrophages for 7 consecutive days (Fig. 5a_1_). Compared to the control group, M1 macrophages in the model group exhibited significantly elevated levels of all three inflammatory cytokines, confirming the successful establishment of the *in vitro* inflammatory model. Notably, cytokine levels were markedly reduced in the J-HD-treated group relative to the model group, indicating the effective suppression of pro-inflammatory cytokine secretion by the J-HD patch (Fig. 5a_2_). These findings align with the 7-day peak therapeutic window for adhesion prevention and demonstrate that the J-HD patch can modulate macrophage-mediated inflammation at the molecular level. This immunoregulatory capacity is a critical prerequisite for its anticipated *in vivo* anti-adhesion performance.

The adhesion of fibrinogen and blood cells marks the initial stage of postoperative adhesion formation. The coagulation cascade, triggered by blood cells, activates thrombin, leading to excessive fibrin deposition, which ultimately facilitates the development of fibrous adhesions [61]. As the condition progresses, the adhesion and aggregation of fibroblasts become crucial in the maturation of adhesive lesions. To evaluate the *in vitro* anti-adhesion performance of the J-HD patch, the top and bottom surfaces were co-incubated with fibrinogen (Fig. 5b), blood cells (Fig. 5c), and L929 fibroblasts (Fig. 5d). Compared to standard tissue culture polystyrene (TCPS), the top HD layer of the J-HD patch demonstrated substantial adhesion to both fibrinogen and blood cells. In contrast, the bottom HD-P_M_ layer exhibited significantly reduced adhesion to both, indicating effective anti-adhesive properties. Similarly, the HD layer supported the attachment and growth of L929 cells comparable to TCPS, whereas the HD-P_M_ layer showed minimal cell adhesion. These differences in bioadhesion are closely correlated with the surface wettability of the layers [62]. Contact angle measurements indicated that the top HD layer was highly hydrophobic (123.30°), whereas the bottom HD-P_M_ layer was hydrophilic (37.27°) (Fig. S2). The hydrophilic HD-P_M_ layer established a stable and tightly bound hydration layer, which functioned as a high-energy physical barrier by anchoring a significant number of water molecules—an essential mechanism known to prevent nonspecific protein adsorption and cellular attachment [38]. Furthermore, the crosslinking of HD and PF127 with PEGDE consumed a substantial number of internal hydrogen bonds, resulting in a denser polymer network that further reduced the number of active adhesion sites on the film surface [63]. Meanwhile, the HD layer exhibited unique dual functionality: its hydrophobic methyl groups contributed to surface water repellency, while its abundant hydrogen bonding capacity and topological chain entanglements facilitated cell and protein adhesion [36].

### 3.4 *In vivo* anti-adhesion efficacy

**Fig. 6:**
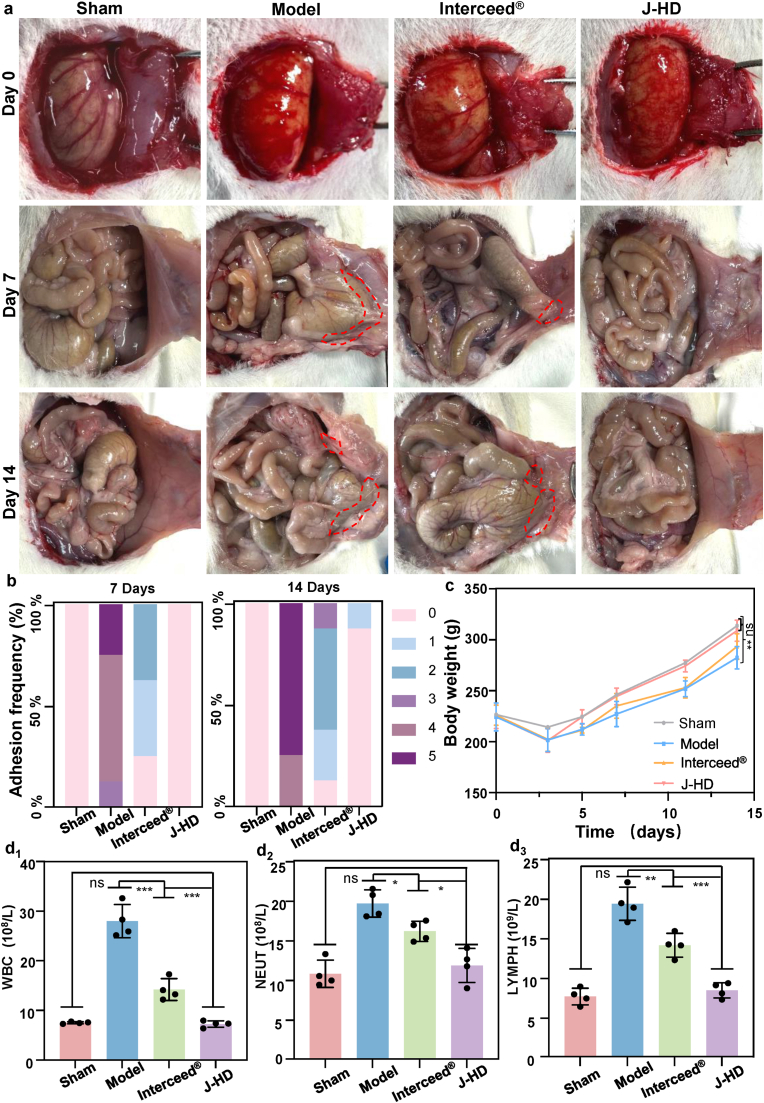
*In vivo* evaluation of the postoperative anti-adhesion efficacy following the application of the J-HD patch. (a) Representative photographs illustrating the adhesions in each group on postoperative days 7 and 14. (b) Adhesion scores on postoperative days 7 and 14 (n = 8). (c) Postoperative changes in the body weight of the rats over time (n = 8). WBC counts (d_1_) NEUT (d_2_) levels, and LYMPH (d_3_) levels for each group on postoperative day 14 (n = 4). Statistical significance was determined by one-way ANOVA with ∗*p* < 0.05, ∗∗*p* < 0.01 and ∗∗∗*p* < 0.001. Error bars show standard deviation, ns indicates no significant difference.

The *in vivo* anti-adhesion efficacy of J-HD patches was evaluated using a rat sidewall defect–cecal abrasion model. Rats were randomly assigned to six groups: the Sham group (abdominal incision and closure without injury), the model group (saline-treated), the Interceed® group (commercial anti-adhesion barrier), and the J-HD group (Fig. 6a). Given that postoperative adhesions typically begin to form within 5–7 days and become irreversible by 7–14 days, adhesion status was assessed at both time points. Adhesion areas were outlined with red dashed lines, and severity was graded using a standardized adhesion scoring system (Fig. 6b). Severe adhesions were observed in the model group on days 7 and 14, with average scores of 4.13 and 4.75, respectively. In contrast, no adhesions were found in the Sham group, confirming that surgery and suturing alone do not typically lead to adhesion formation. The Interceed® group showed slightly improved outcomes compared to the model group, with average scores of 1.13 and 1.63 on days 7 and 14, respectively. While Interceed® provides a degree of mechanical strength and serves as a physical isolation barrier resistant to abdominal pressure and shear forces, its inability to modulate the wound healing process limits its overall efficacy in preventing adhesions. In contrast, the J-HD group exhibited the most effective anti-adhesion performance, with mean adhesion scores of 0 and 0.125 on days 7 and 14, respectively. The J-HD patch effectively prevented adhesion formation, likely due to its integrated functions: serving as a robust physical barrier, actively inhibiting fibroblast and fibronectin adhesion, and promoting favorable wound healing. Nearly all rats in the J-HD group displayed complete healing without visible adhesions.

In addition, the postoperative weight gain curves of rats in the J-HD group showed no significant differences compared to those in the Sham group (Fig. 6c). This indicates that J-HD treatment may facilitate the recovery of intestinal function, which is positively correlated with its strong anti-adhesion properties. To further evaluate the systemic inflammatory response, we measured blood levels of white blood cells (WBC), neutrophils (NEUT), and lymphocytes (LYMPH). As illustrated in Fig. 6d_1_–d_3_, the levels of WBC, NEUT, and LYMPH in the J-HD group were comparable to those in the Sham group on day 14, indicating an effective resolution of inflammation. Additionally, plasma levels of the pro-inflammatory cytokines TNF-α and IL-6 were quantified using ELISA (Fig. S3). Compared to the model group, the J-HD group exhibited significantly reduced levels of both cytokines, with no significant differences observed in relation to the Sham group.

**Fig. 7:**
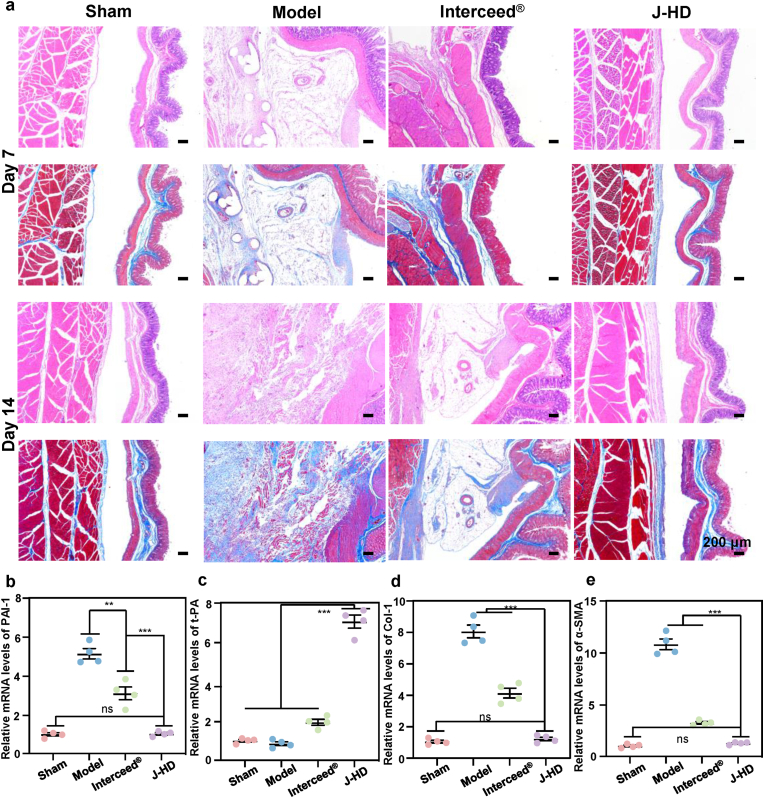
*In vivo* histological and gene expression analysis of adhesion tissues. (a) H&E staining and Masson's trichrome staining of typical adhesion tissues on postoperative days 7 and 14. (b–e) Relative mRNA levels of (b) plasminogen activator inhibitor-1 (PAI-1), (c) tissue plasminogen activator (t-PA), (d) collagen type I (Col-1) and (e) α-smooth muscle actin (α-SMA) in injured tissues (n = 4). Statistical significance was determined by one-way ANOVA with ∗∗*p* < 0.01 and ∗∗∗*p* < 0.001. Error bars show standard deviation, ns indicates no significant difference.

Abnormal collagen deposition and damage in the cecum and abdominal wall were observed and evaluated using H&E staining and Masson's trichrome staining on days 7 and 14 (Fig. 7a). On postoperative day 7, significant adhesions were present in the model group, and irreversible deep tissue adhesions were noted on day 14. The boundary between the visceral peritoneum and the abdominal wall peritoneum became indistinct, accompanied by disorganized cellular arrangements and marked proliferation of fibrous connective tissue. Concurrently, H&E staining revealed extensive infiltration of inflammatory cells, while Masson's staining indicated substantial collagen deposition. The mesothelium of both the cecum and abdominal wall exhibited significant damage. Abnormal collagen deposition and inflammatory cell infiltration were also observed in the plasma layer of the cecum and the abdominal wall of the Interceed® group on the 7th day. Furthermore, the adhesions between the two groups worsened by the 14th day. This exacerbation can be attributed to the fact that, although Interceed® possesses inherent anti-adhesion properties that can reduce postoperative adhesions to some extent, it is still unable to completely prevent the formation of adhesions. This limitation arises from the Interceed® film's lack of intrinsic tissue adhesion properties, which hinders its ability to prevent adhesion failure due to displacement. In contrast, this phenomenon was not observed in the J-HD group, which resembled the Sham group. This difference may be due to the self-leveling J-HD patch providing superior wound coverage during the early stages of adhesion progression. Additionally, the unique tissue adhesion properties of the J-HD patch prevented displacement, thereby ensuring its effectiveness in preventing the development of adhesions during the initial stages of adhesion formation. The J-HD patch can maintain a stable physical barrier throughout the adhesion formation process without impeding wound healing.

RT-PCR was conducted to evaluate the mRNA transcript levels of PAI-1, t-PA, Col-1 and α-SMA, in injured tissues at the critical time point of day 7. The cytokines t-PA and PAI-1 play essential roles in regulating fibrinolysis *in vivo*. Disruption of the fibrinolytic balance results in abnormal collagen deposition, primarily Col-1, which subsequently promotes the formation of adhesions [64]. Furthermore, the myofibroblast is a key cell type involved in adhesion formation; it produces increased amounts of fibrillar collagen and other matrix proteins and express α-SMA, a molecular marker of activated myofibroblasts [38].Compared to the model and Interceed® groups, the relative mRNA expression levels of PAI-1 and Col-1 were significantly decreased in the J-HD group, while the relative mRNA expression of t-PA was significantly increased, indicating that the fibrinolytic balance was restored (Fig. 7b–d). Additionally, the relative mRNA expression level of α-SMA in the J-HD group was significantly lower compared to those in the model and Interceed® groups (Fig. 7e). This further confirms the effectiveness of the J-HD patch in preventing the deposition of aberrant collagen by shifting the fibrillar equilibrium towards fibrinolysis, thereby exerting anti-adhesion effects.

### 3.5 Repair of intestinal wall integrity by J-HD patch

**Fig. 8:**
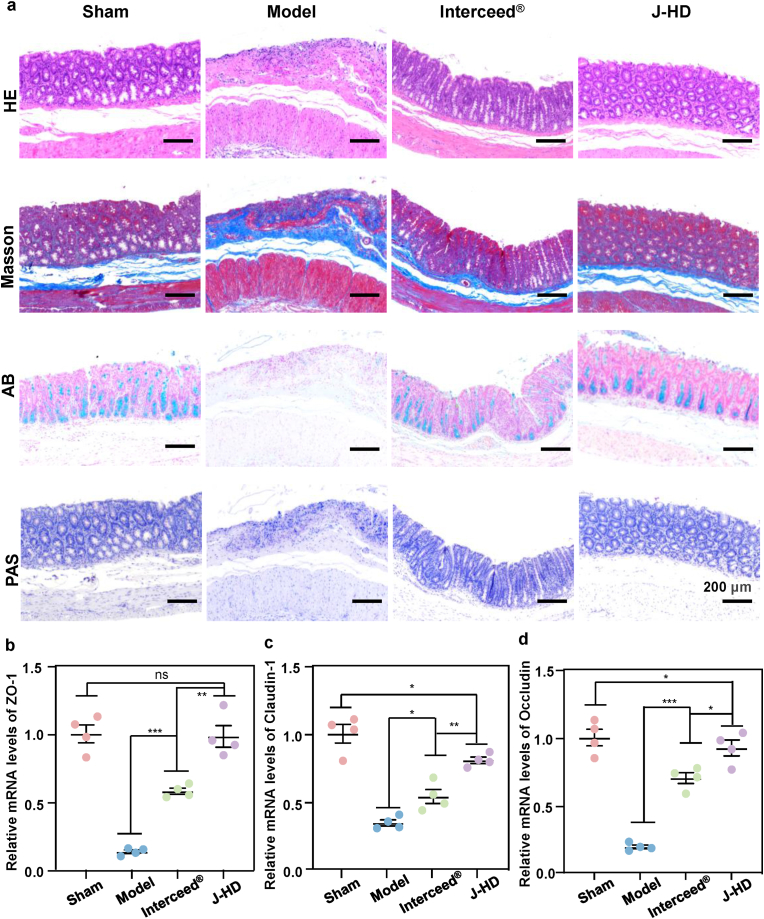
Repair of intestinal wall integrity using the J-HD patch on postoperative Day 7. (a) Representative images of injured rat cecum tissues stained with H&E, Masson's trichrome, Alcian blue (AB), and Periodic Acid-Schiff (PAS) stain. mRNA transcription levels of tight junction proteins, including (b) ZO-1, (c) Occludin, and (d) Claudin-1 (n = 4). Statistical significance was determined by one-way ANOVA with ∗∗*p* < 0.01 and ∗∗∗*p* < 0.001. Error bars show standard deviation, ns indicates no significant difference. (For interpretation of the references to colour in this figure legend, the reader is referred to the Web version of this article.)

Disruption of intestinal integrity following abdominal surgery exacerbates postoperative intestinal dysfunction and induces adhesions [65]. H&E staining and Masson trichrome staining revealed significant signs of severe mucosal disruption, extensive infiltration of inflammatory cells, loss of crypts, and collagen deposition in the damaged cecal mucosa of the model group. In contrast, the J-HD group exhibited a notable increase in cisternae between the epithelial cells of the injured cecal mucosa and the epithelial cells of the intestinal glands, accompanied by a decrease in collagen deposition (Fig. 8a). These findings suggest that J-HD patches can effectively restore the integrity of the intestinal wall. The intestinal mucus barrier is primarily composed of mucus secreted by goblet cells, which serves to isolate the intestinal flora from host cells. This mechanism effectively prevents excessive inflammatory responses in the intestine and acts as the first line of defense, protecting the intestinal epithelium from invasion [66]. AB staining and PAS staining revealed a significant loss of goblet cells and mucus in the model group, while the J-HD group exhibited results similar to the Sham group, with both the number and structure of goblet cells well-preserved. This preservation may be attributed to the anti-inflammatory properties of the J-HD patch, which effectively reduces inflammation in the injured cecum region, thereby preventing disruption of the epithelial barrier and inhibiting inflammation-related fibrous deposition. In comparison to the Interceed® group, the J-HD patch **exhibited superior efficacy in restoring** intestinal wall integrity and **rejuvenating goblet cell** function. This enhanced performance is likely attributable to **its self-leveling property and immediate unilateral adhesion**, which facilitate tissue retention, **as well as** its degradation profile **that is synchronized with** the wound healing process.

Tight junction proteins, including Claudin-1, ZO-1, and Occludin, play a crucial role in maintaining intestinal homeostasis [67]. In terms of molecular mechanisms, Claudin-1 serves as a major structural component of epithelial intercellular junctions, while ZO-1 anchors tight junctions to the cytoskeleton of epithelial and endothelial cells. Occludin functions as a core scaffolding protein that regulates the architecture of intercellular junctions. These proteins work synergistically to regulate cellular permeability by stabilizing the junction complex, thereby preserving intestinal integrity and physiological function [68]. RT-PCR assays have revealed differential mRNA expression patterns of these tight junction markers (Fig. 8b–d). Although both physical barrier interventions significantly upregulated the transcript levels of the three proteins, the Interceed® group did not achieve optimal results due to its insufficient *in situ* retention capacity. Notably, there was no statistically significant difference in ZO-1 mRNA expression between the J-HD group and the Sham group, which may be attributed to the higher sensitivity of ZO-1 to inflammatory regulation compared to other connexins [69]. The present study demonstrated that the J-HD patch offers dual therapeutic advantages: 1) it physically isolates the injured cecum tissue from the abdominal wall, and 2) it promotes intestinal barrier repair through the upregulation of tight junction proteins. This mechanism of action may effectively inhibit the inflammation-related fibrotic deposition process by maintaining structural integrity and normal barrier function [70].

### 3.6 Immunomodulatory role of J-HD patches

**Fig. 9:**
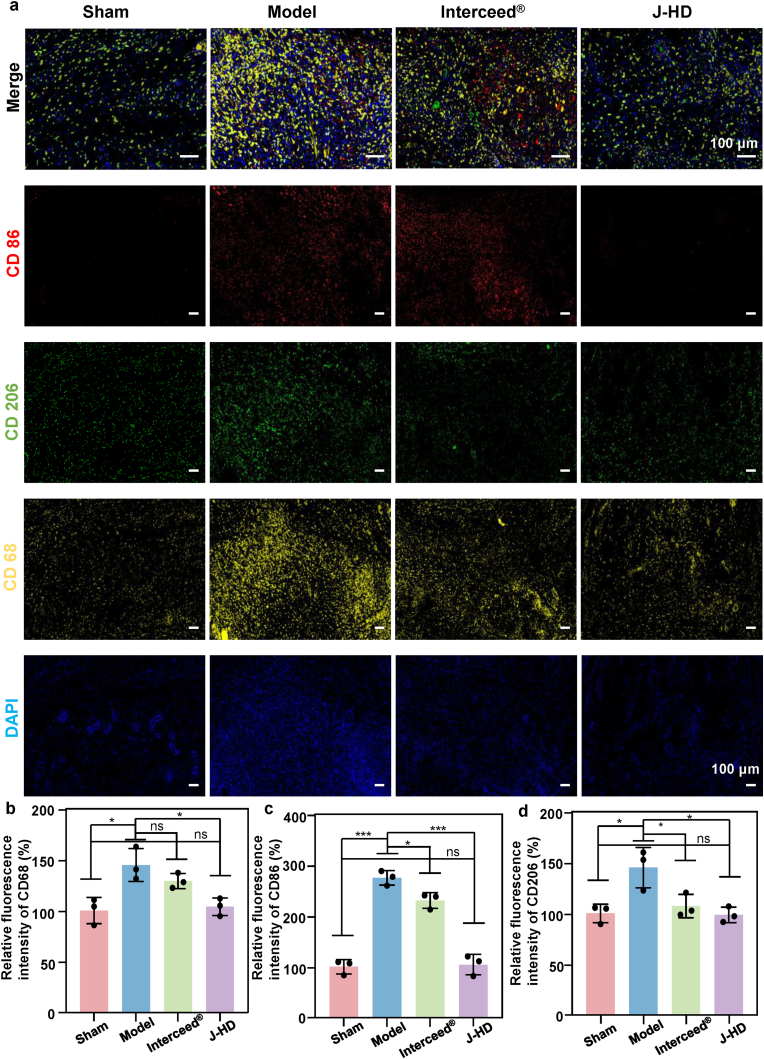
Immunomodulatory effect of the J-HD patch on postoperative Day 7. (a) Merged immunofluorescence staining image of injured rat cecum tissue. (b) Immunofluorescence staining of CD86 (red), CD206 (green), CD68 (yellow), and DAPI (blue) in injured rat cecum tissues. Relative quantitative analysis of (b) total macrophages, (c) M1 macrophages, and (d) M2 macrophages (n = 3). Statistical significance was determined using one-way ANOVA with ∗*p* < 0.05 and ∗∗∗*p* < 0.001. Error bars show standard deviation, ns indicates no significant difference. (For interpretation of the references to colour in this figure legend, the reader is referred to the Web version of this article.)

In the abdominal wall-intestinal wall composite injury model, the local microenvironment triggers the recruitment and activation of macrophages [71]. These immune cells undergo functional polarization, differentiating into a pro-inflammatory M1 phenotype (CD86) and a pro-fibrotic M2 phenotype (CD206). Macrophages (CD68), M1 (CD86), and M2 (CD206) were qualitatively assessed through specific immunofluorescence analysis on postoperative day 7, a critical stage for adhesion formation (Fig. 9a). The results indicated that the overall level of macrophage infiltration in the J-HD group was significantly lower than that in the model group (Fig. 9b). This finding confirms that the potent anti-adhesion efficacy of J-HD patches is partly attributable to their crucial role in regulating the balance of macrophage polarization. The reduction of M1 macrophages (CD86) in the J-HD group suggests a decrease in the inflammatory response (Fig. 9c), while the decrease in M2 macrophages (CD206) helps prevent excessive fibrocollagen deposition. This synergistic down-regulation of M1 and M2 macrophages significantly reduces the risk of aberrant fibrillar collagen deposition in the J-HD group, and the reduction of M2 macrophages (CD206) further mitigates excessive fibrillar collagen accumulation (Fig. 9d).

### 3.7 Biocompatibility and host response assessment

The evaluation of cytotoxicity, hemolytic properties, and host response of the J-HD patch was conducted to assess its biocompatibility following subcutaneous implantation. Live/dead staining of L929 cells demonstrated no significant cell death after 24, 48, and 72 h of co-incubation with the J-HD patch, with most cell boundaries appearing clear, intact, and outwardly extended. In conclusion, the high cell viability and well-defined cell morphology indicate that the J-HD patch is safe for cell survival (Fig. S4). The quantitative cell counting kit assay showed that cell viability across all concentrations of J-HD patch treatment was comparable to that of the control treatment, indicating that the J-HD patch is non-toxic (Fig. S5). In comparison to the negative control, the hemolysis rate of the J-HD patch was approximately 0.17 %, significantly lower than the safety threshold of 5 % (Fig. S6) [72]. This finding suggests that the J-HD patch possesses excellent blood compatibility, is safe and non-toxic, and can be utilized as a biomedical material.

An evaluation of the host response in rats following subcutaneous implantation of the J-HD patch revealed no significant differences in blood parameters (Table S3) when compared to the control group (unimplanted rats). Throughout the degradation process, no signs of congestion, redness, or inflammation were observed at the implantation site. Correspondingly, H&E and Masson's staining of the tissues at the implantation site revealed no significant infiltration of inflammatory cells or abnormal collagen deposition (Fig. S7). Additionally, the major organs—including the heart, liver, spleen, lungs, and kidneys—showed no signs of damage (Fig. S8). Collectively, these results indicate that J-HD patch implantation does not exhibit significant acute or chronic toxicity *in vivo*, elicits an ideal host response post-implantation, and demonstrates appropriate degradation properties, along with excellent biocompatibility and an effective anti-adhesion process.

### 3.8 Interaction of comprehensive anti-adhesion strategies

**Fig. 10:**
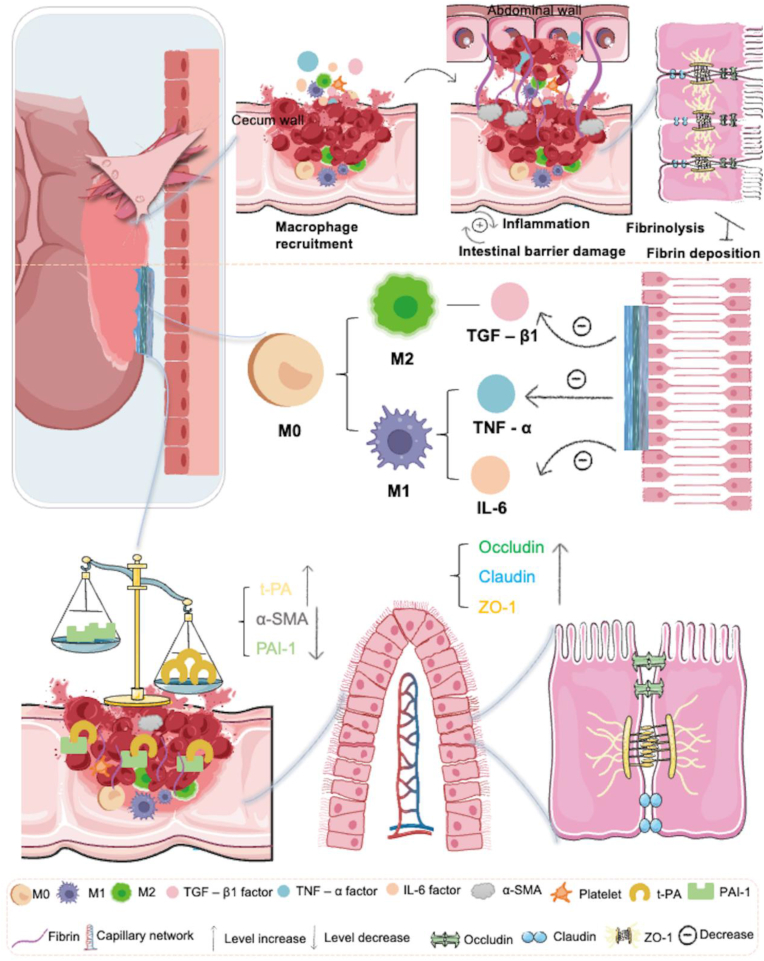
The “three-in-one” anti-adhesion mechanism of the J-HD patch includes maintaining intestinal barrier integrity, regulating fibrosis balance, and reducing the binding of inflammatory factors.

J-HD patches create a self-enhancing effect through three synergistic anti-adhesion mechanisms: structural maintenance, fibrinolytic modulation, and immune homeostasis. This process promotes trauma repair while multidimensionally inhibiting adhesion pathology (Fig. 10). Among these mechanisms, the reconstruction of local immune homeostasis is central to the regulatory network. Surgical injury initiates a coagulation-inflammation cascade; thrombin released during tissue hemostasis simultaneously activates the fibrinogenic system (mediating the conversion of fibrinogen via t-PA) and the immune response system (polarizing resident and migrating macrophages toward pro-inflammatory M1 phenotypes). This ultimately results in a persistent inflammatory infiltrate [62]. Activated M1 macrophages exacerbate adhesion formation by: 1) secreting pro-inflammatory mediators (such as IL-6 and TNF-α) to enhance inflammatory signaling, and 2) upregulating PAI-1 expression to inhibit t-PA activity, thereby establishing a procoagulant-antifibrinolytic microenvironment [63].

This pathological cascade was systematically regulated by J-HD patch intervention. First, the overexpression of pro-inflammatory cytokines IL-6 and TNF-α was inhibited to eliminate the abnormal deposition of Col-1; secondly, the production of PAI-1, and α-SMA was suppressed, while the release of t-PA was increased to alleviate local inflammation. This process accelerated fibrinolysis, thereby shifting the balance towards non-adhesion. By reducing the local inflammatory response and strengthening intestinal cellular junctions, the intervention disrupts the vicious cycle of worsening inflammation and damage to intestinal structures. In conclusion, the J-HD patch complements itself in three key aspects: maintaining intestinal structural integrity, regulating fibrinolytic homeostasis, and establishing a positive feedback loop for anti-inflammatory immune responses. Collectively, these mechanisms effectively prevent postoperative intra-abdominal adhesions. Furthermore, the vicious cycle of infiltration and intestinal barrier disruption is interrupted by enhancing the stability of ZO-1, a tight junction protein complex. This triad of mechanisms—maintenance of the mucosal repair microenvironment, reestablishment of fibrinolytic homeostasis, and creation of an anti-inflammatory immune feedback loop—synergistically enhances the anti-adhesion effect at the systems biology level.

## 4 Conclusion

We have developed a three-layer biodegradable J-HD patch, composed of HD, PVA, and HD-PF127 from top to bottom, that exhibits strong wet tissue adhesion, favorable mechanical properties, and effective anti-post-operative adhesion. This innovative patch addresses the challenges associated with previous anti-adhesive materials, such as the dislocation of anti-adhesive agents, non-specific tissue adhesion, and the induction of secondary fibrinolytic disorders. It achieves this by combining robust wet tissue adhesion — facilitated through hydrogen bonding and topological adhesion — with self-leveling properties that adapt to irregular wound surfaces. The patch exhibits instantaneous unilateral adhesion and stable interfacial integration, allowing it to firmly adhere to the surface of wet tissue defects. Conversely, the opposite side demonstrates excellent resistance to cell and fibrin adhesion, thereby preventing secondary tissue damage and membrane migration. This innovative design effectively overcomes the limitations of existing anti-adhesion membranes, which often require suturing and fixation. Comprehensive analyses at both the molecular and tissue levels reveal that the J-HD patch modulates fibrinolytic homeostasis, inhibits autoinflammatory responses and restores intestinal wall integrity, ultimately achieving a remarkably significant anti-adhesion effect within 14 days. This multifactorial strategy addresses the need for a physical barrier, maintains fibrinolytic homeostasis, and controls the inflammatory cascade. *In vivo* experiments demonstrated that J-HD patches exhibited neither acute nor chronic toxicity, indicating good histocompatibility and significant clinical translational potential. This provides a viable strategy for developing a new generation of degradable anti-adhesion materials.

## CRediT authorship contribution statement

**Rui Fang:** Writing – original draft, Software, Methodology, Investigation, Formal analysis, Data curation, Conceptualization. **Ning Yu:** Resources, Investigation, Formal analysis. **Simeng Chen:** Validation, Resources, Methodology. **Xi Xu:** Visualization, Validation, Project administration. **Jianfa Zhang:** Writing – review & editing, Supervision, Project administration, Funding acquisition.

## Declaration of competing interest

The authors declare that they have no known competing financial interests or personal relationships that could have appeared to influence the work reported in this paper.

## Data Availability

Data will be made available on request.
