# Supplementary material for: Hemoadhican-based self-leveling Janus patch for comprehensive prevention of postoperative adhesions
Source: Mater Today Bio. 2025 Jun 24;33:102021. doi: 10.1016/j.mtbio.2025.102021 (PMC12264631; doi:10.1016/j.mtbio.2025.102021)
Supplement: Multimedia component 1 [file mmc1.docx]

Supporting Information

Hemoadican-Based Self-Leveling Janus Patch for Comprehensive Prevention of Postoperative Adhesions

Rui Fang ^a,b^, Ning Yu ^a,b^, Xi Xu ^a,b^, Jianfa Zhang ^a,b *^

^a^ Center for Molecular Metabolism, Nanjing University of Science & Technology

^b^ Key laboratory of Metabolic Engineering and Biosynthesis Technology, Ministry of industry and information technology

Nanjing University of Science & Technology

Nanjing 210094, China

mail: [jfzhang@mail.njust.edu.cn](mailto:jfzhang@mail.njust.edu.cn)

**Supplementary Experimental Results**

**Table S1.** Double-blind scoring of tissue adhesions following a standard scoring system.

| Score | Events or phenomena |
| --- | --- |
| 0 | A smooth and intact surface without adhesion occurrence |
| 1 | A single-layer thin-film adhesion on the surface of the cecum |
| 2 | Multiple layers of thin-film adhesion present on the surface of the cecum |
| 3 | Localized adhesion on the cecum exceeding the thickness of the thin film |
| 4 | Multiple thick adhesions with localized adhesion attached to the surface of the cecum |
| 5 | The thick adhesion formed between the cecum and surrounding tissues has reached a vascularized degree |

**Table** **S2.** The sequences of primers used in RT-qPCR

| Primer | Sequence (5’-3’) |
| --- | --- |
| PAI-1 (forward primer) | CGGCACAATCCAACAGAGAC |
| PAI-1 (reverse primer) | ACACGTCCAGTTTTGTCCCA |
| t-PA (forward primer) | GGAAAGAAGCAAGCAAGGCAC |
| t-PA (reverse primer) | GAACCTCCTGTGTATTCCCTGG |
| Col-1 (forward primer) | CGTGGAAACCTGATGTATGCTTG |
| Col-1 (reverse primer) | CCTATGACTTCTGCGTCTGGTGA |
| α-SMA (forward primer) | AGGGAGTGATGGTTGGAATGGG |
| α-SMA (reverse primer) | GGTGATGATGCCGTGTTCTATCG |
| TGF-β3 (forward primer) | CCTGGCCCTGCTGAACTTG |
| TGF-β3 (reverse primer) | TTGATGTGGCCGAAGTCCAAC |
| TGF-β1 (forward primer) | CATTGCTGTCCCGTGCAGA |
| TGF-β1 (reverse primer) | AGGTAACGCCAGGAATTGTTGCTA |
| ZO-1 (forward primer) | GCCAGCTTTAAGCCTCCAGA |
| ZO-1 (reverse primer) | TGGCTTCGCTTGAGGTTTCT |
| Claudin-1 (forward primer) | TGTGTCCACCATTGGCATGA |
| Claudin-1 (reverse primer) | ACTAATGTCGCCAGACCTGAAA |
| Occludin (forward primer) | GGGGCGCAGCAGGTCT |
| Occludin (reverse primer) | GCCTGTAAGGAGGTGGACTC |
| β-actin (forward primer) | GATATCGCTGCGCTCGTC |
| β-actin (reverse primer) | TGGGGTACTTCAGGGTCAGG |


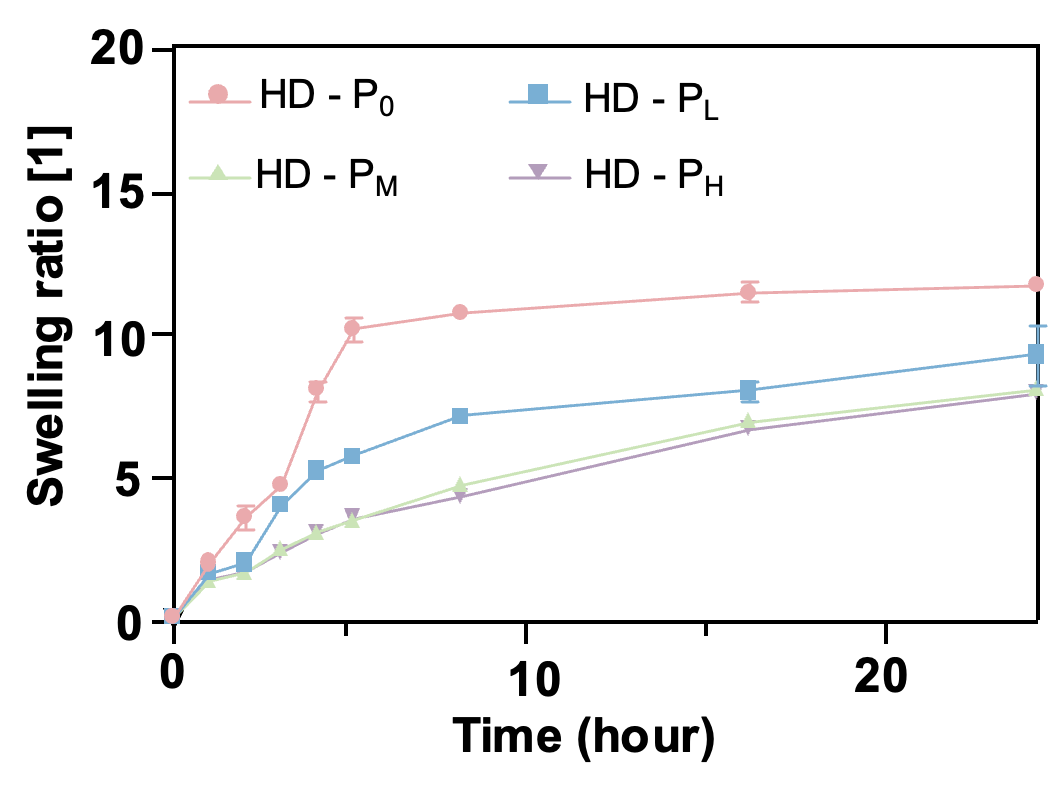


**Fig. S1.** 24-hour swelling rate of HD-P film (n=3).

**HD**

***


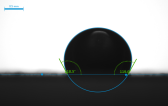

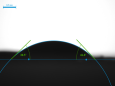


**Contact angle (°)**

**HD-P**

**Fig. S2.** Water contact angle (WCA) tested by a sessile-drop method (n=3). Statistical significance was determined by Student’s *t*-test with ****p* < 0.001. Error bars show standard deviation.


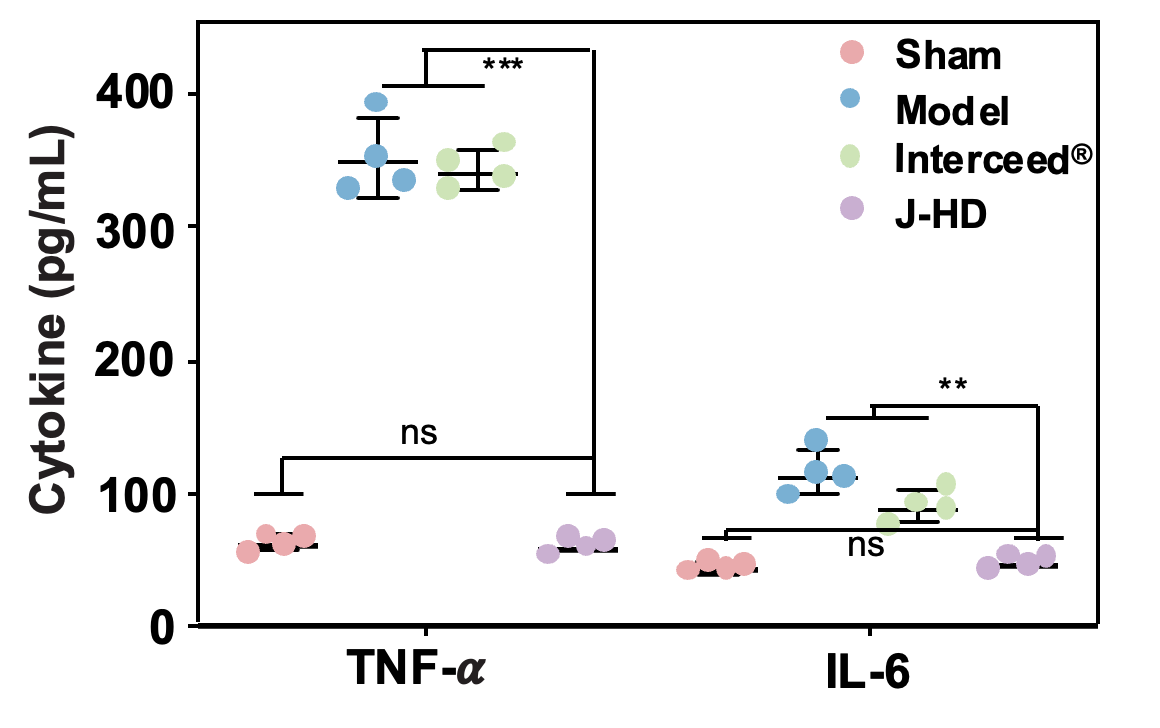


**Fig. S3.** TNF-α and IL-6 levels of each group on postoperative days 14 (n=4). Statistical significance was determined by one-way ANOVA with***p* < 0.01, ****p* < 0.001. Error bars show standard deviation, ns indicates no significant difference.


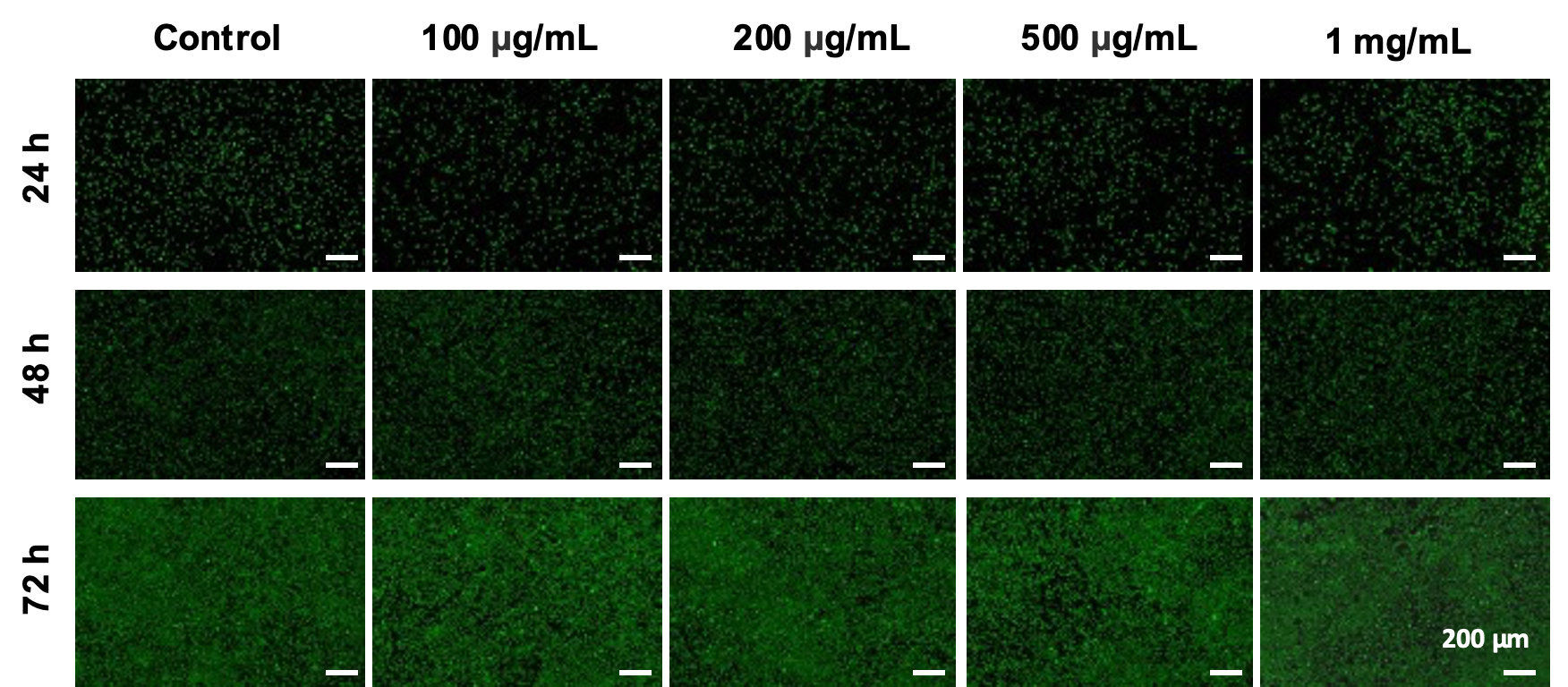


**Fig. S4.** Live/Dead staining of L929 cells after co-incubation with J-HD patch samples for 24-72 h.

**100**

**0**

**50**

**Cell vialibility (%)**

**Control**

**0.1**

**0.2**

**0.5**

**1**

**J-HD concentration (mg/mL)**

**Fig. S5.** Viability of L929 cells treated with different concentrations of J-HD patch samples. (n=4)


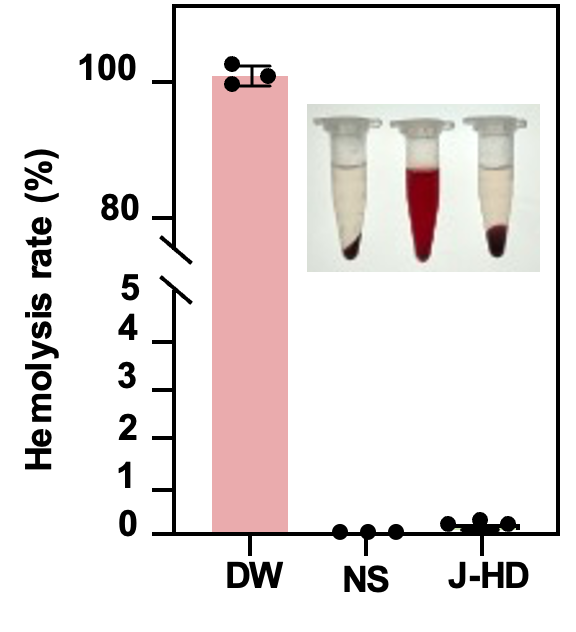


**Fig. S6.** Hemolysis assay of J-HD treatment (n=3).

**Table S3.** Hematological and blood biochemical analyses in rats after the subcutaneous implantation of J-HD patch

| Index | Wound | J-HD |
| --- | --- | --- |
| WBC (10^9^/L) | 7.12 ± 0.85 | 6.82 ± 0.73 |
| RBC (10^12^/L) | 7.37±0.87 | 8.07 ± 0.59 |
| HGB(g/L) | 150.67 ± 18.01 | 130.33 ± 16.50 |
| HCT (%) | 52.7±3.83 | 43.65 ± 6.87 |
| PLT (10^11^/L) | 435.8±39.64 | 432.93 ± 58.81 |
| LYMPH (10^9^/L) | 3.03±0.90 | 3.43 ± 0.39 |
| NEUT (10^8^/L) | 5.75±0.77 | 5.88 ± 0.48 |
| MONO (10^9^/L) | 2.37±0.85 | 2.97 ± 1.01 |


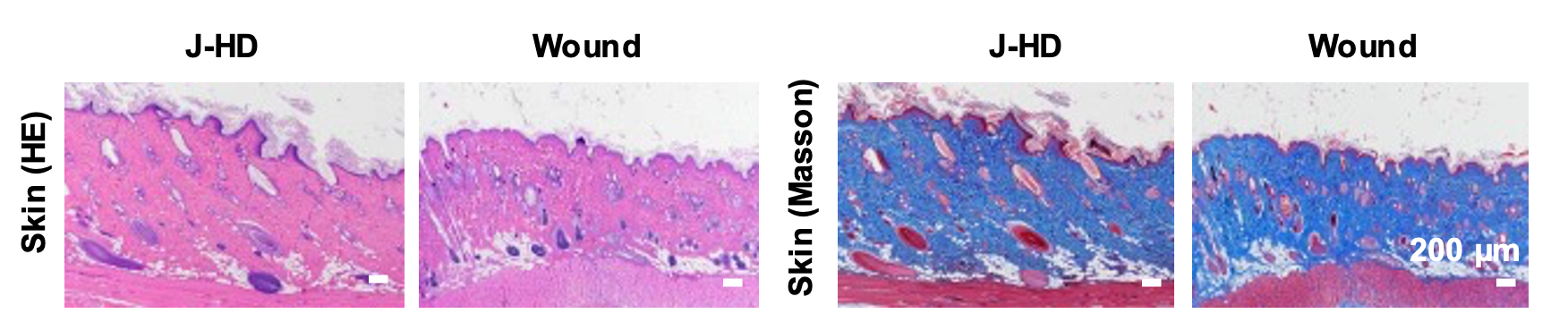


**Fig. S7.** The histological changes of stained sections of skin.


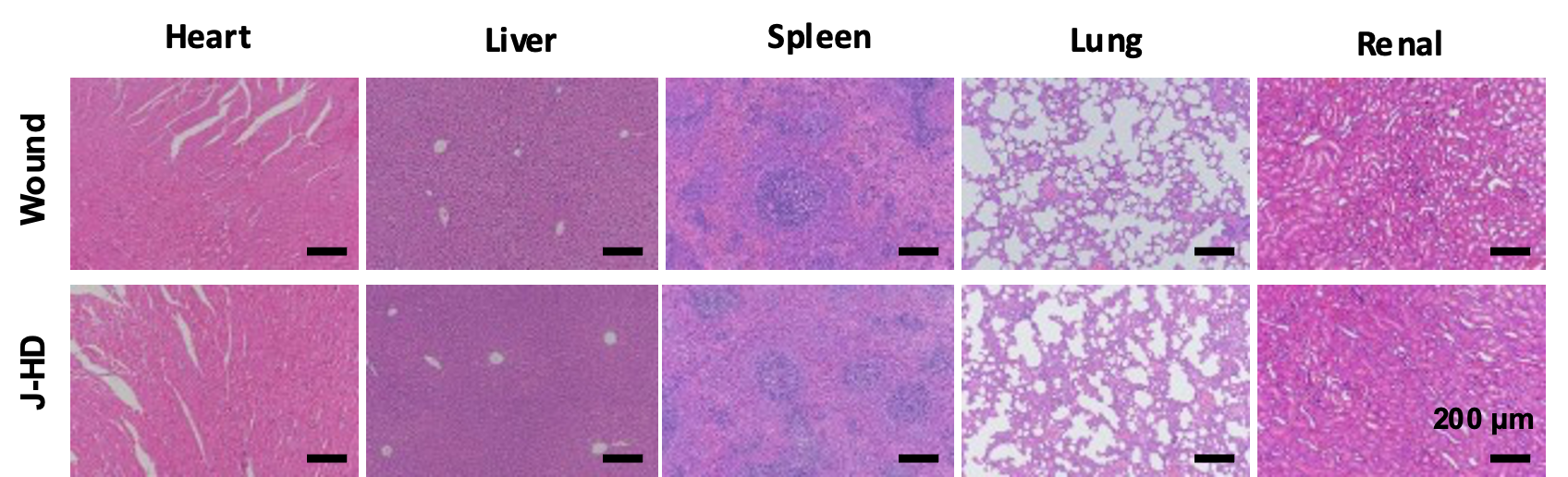


**Fig. S8.** H&E staining and Masson’s trichrome staining of heart, liver, spleen, lung and kidney.
